# Microwave Ablation Versus Wedge Resection for Stage I Non-small Cell Lung Cancer Adjacent to the Pericardium: Propensity Score Analyses of Long-term Outcomes

**DOI:** 10.1007/s00270-020-02601-7

**Published:** 2020-09-09

**Authors:** Hao Hu, Bo Zhai, Rong Liu, Jia chang Chi

**Affiliations:** 1 Department of Interventional Oncology, Renji Hospital, School of Medicine, Shanghai Jiao Tong University, Shanghai, China; 2 Department of Interventional Radiology, Zhongshan Hospital, Shanghai Medical College, Fudan University, Shanghai, China

## Abstract

**Objective:**

The present study has compared the long-term outcomes between performing wedge resection (WR) and microwave ablation (MWA) as first-line treatment of stage I non–small cell lung cancer (NSCLC) patients with tumors adjacent to the pericardium.

**Materials and Methods:**

Between January 2014 and December 2018, a total of 223 consecutive patients with T1N0 NSCLC underwent first-line treatment by WR (*n* = 155) or image-guided lung MWA (*n* = 68). This study has compared the progression-free survival (PFS) and overall survival (OS) rates between the two treatments before and after propensity score matching. Subgroup analysis of these outcomes was conducted based on the distance from the pericardium.

**Results:**

The median follow-up time was 47 months. Propensity matching yielded 56 pairs of patients. In the two matched groups, the PFS rates in the WR group at 3 and 5 years were 66.0% and 56.0% and 54.0% and 36.0%, respectively, in the MWA group (*P* = 0.029). Meanwhile, the corresponding OS rates for the WR group at 3 and 5 years were 81.0% and 72.0% and 60.0% and 55.0% in the MWA group, respectively (*P* = 0.031). Subgroup analysis, done according to the treatment modality, indicated that local tumor recurrence and PFS for NSCLCs that were close but not contiguous to the pericardium were different from those contiguous to the pericardium (*P* = 0.018 and *P* = 0.025, respectively).

**Conclusion:**

WR provided better long-term tumor control and OS compared to MWA for stage I NSCLC adjacent to the pericardium as a first-line treatment. MWA can be considered as an alternative option for high-risk and inoperable patients, particularly for tumors that were not contiguous to the pericardium.

## Introduction

Lung cancer accounts for the highest number of cancer-related deaths globally [1]. Anatomic lung resection represents the standard curative therapy for patients with early-stage non–small cell lung cancer (NSCLC). However, approximately 30% of patients are not eligible for surgery because of various reasons, including detection at an advanced-stage, medical comorbidities, insufficient cardiopulmonary function, or poor performance scores [2]. One minimal invasive therapy is percutaneous microwave ablation (MWA), which has emerged as the preferred therapeutic strategy for patients who are not to undergo operation [3, 4]. MWA is minimally invasive, as it only causes mild deleterious effects on pulmonary function, needs a short reconvalescence, and allows for repeated procedures [5]. Several studies have suggested that the outcomes of MWA are equivalent to those of wedge resection (WR) for patients with medical comorbidities and insufficient cardiopulmonary function, especially for those with stage T1N0 [6, 7].

In contrast to WR by the thoracoscopic or open approach, tumor location significantly influences the outcomes of MWA [8, 9]. Therefore, high-risk locations of NSCLC, such as adjacent to the pericardium or large blood vessels, can affect the treatment outcomes after MWA [10–12]. There are controversies on local tumor control by MWA for lung tumors adjacent to the pericardium. Firstly, though difficult, precise antenna placement near vital mediastinal structures is essential to avoid complications arising from puncture or ablation of non-target tissues [13]. In addition, the creation of ablation zones near the heart may cause unpredictable effects due to severe perfusion-mediated convective heat loss created by the heart and large pulmonary vessels [11, 13, 14]. This effect could limit intra-tumor temperatures and result in narrower ablation margins and increased risk of local tumor progression. Besides, ablations performed extremely close to the heart may damage cardiac tissue or trigger dangerous arrhythmias [15].

However, comparative studies on the therapeutic outcomes of WR and MWA for stage I NSCLC adjacent to the pericardium have not been performed. Also, results of previous retrospective studies [16, 17] have indicated that, relative to surgical candidates, patients who undergo MWA are more likely to be older and have medical comorbidities and insufficient cardiopulmonary function, which could affect the long-term outcomes of each treatment.

Thus, the present study conducted a propensity score matching analysis to retrospectively compare the long-term therapeutic outcomes of WR and MWA as a first-line treatment for stage I NSCLC adjacent to the pericardium.

## Materials and Methods

### Patients

**Fig. 1:**
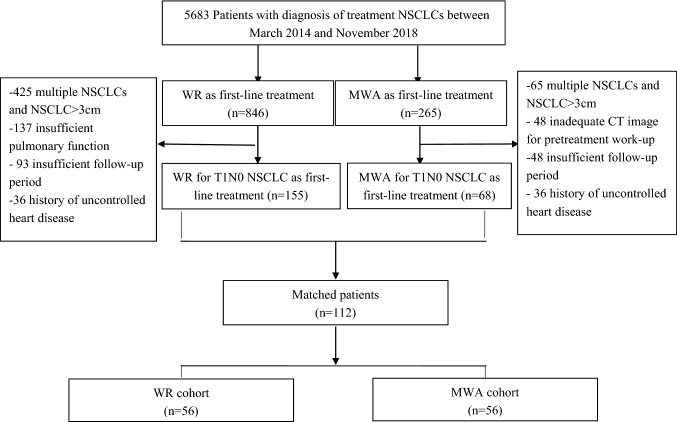
Flow diagram of patient selection for the study

This comparative study was conducted as a retrospective analysis at a hospital affiliated with a tertiary academic institution. The study was approved by the institutional review board, which also waived the requirements for informed consent. Between March 2014 and November 2018, 5683 consecutive patients were diagnosed with NSCLC at our hospital. Out of these patients, 846 underwent WR, while 265 received CT-guided MWA as a first-line treatment. The inclusion criteria study were (1) a small solitary stage I NSCLC (T1a/bN0M0), (2) platelet count ≥ 50 × 10^9^/L, (3) prothrombin time ratio ≥ 70%, (4) Eastern Cooperative Oncology Group (ECOG) performance status score ≤ 2, (5) the use of multiphase dynamic computed tomography (CT) for pretreatment assessment, and (6) more than six-month follow-up after treatment. Patient receiving local ablative treatment is those with lung tumors who refused or were considered unable to tolerate surgical resection and radiotherapy by a multidisciplinary team. Patients were classified on the basis of the distance from the pericardium, into tumor lesions adjacent to the pericardium and tumor lesions remote to the pericardium. Finally, we included patients with biopsy-proven stage I NSCLC adjacent to the pericardium (*n* = 68), and these were treated by CT-guided MWA. The WR group included 155 patients with histologically proven NSCLC. The procedure for patient selection is detailed in Fig. 1.

### Definition of Lesion Location

**Fig. 2:**
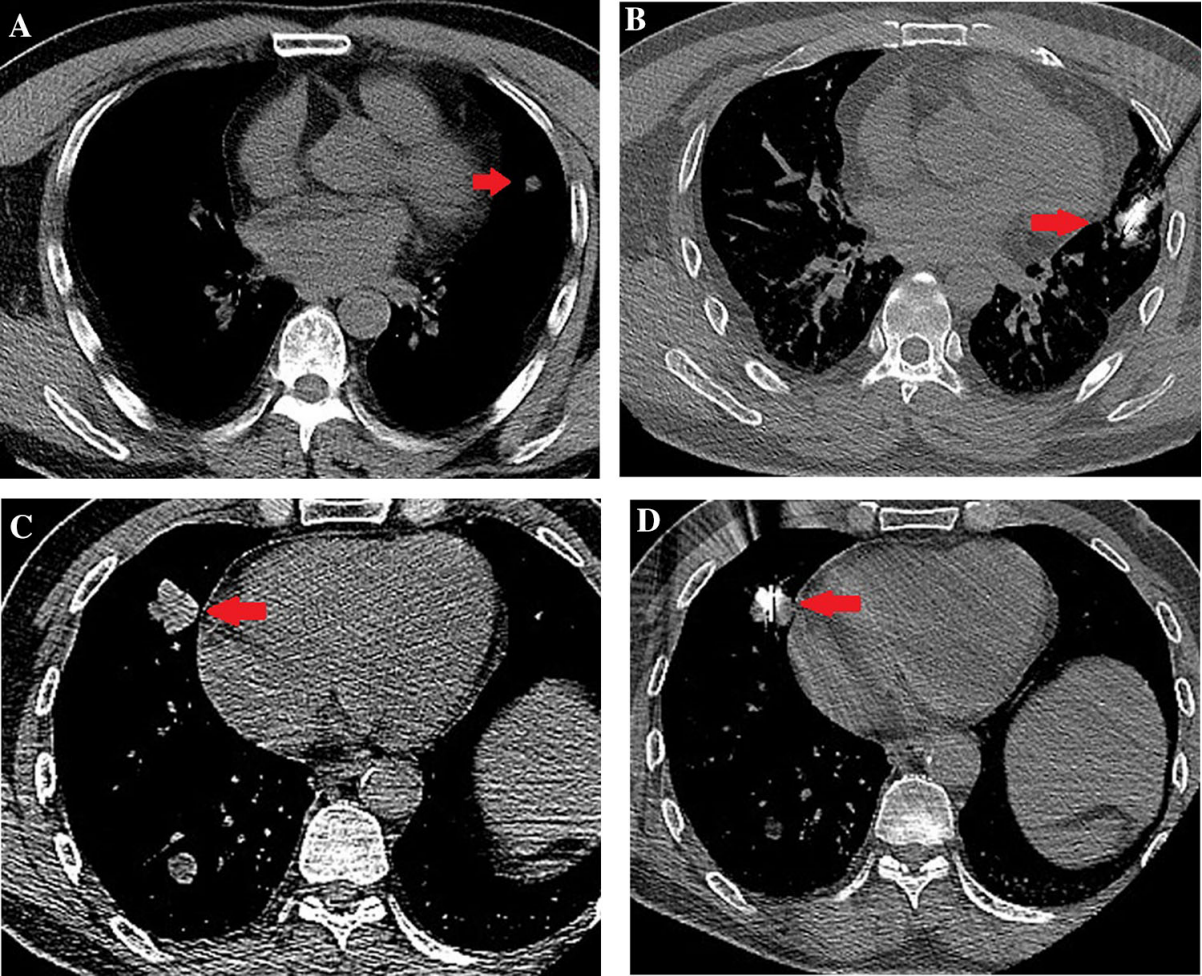
**A** Chest computed tomography (CT) obtained prior to microwave ablation (MWA) shows a 1.2-cm well-defined round tumor (red arrow) in the left lobe (lesions that were not contiguous to the pericardium). **B** In the supine position, MWA was performed on the lung tumor adjacent to the pericardium (red arrow). **C** CT obtained prior to MWA shows a 2.8-cm well-defined round tumor (red arrow) in the right lobe (lesions that were contiguous to the pericardium). **D** In the supine position, MWA was performed on the lung tumor adjacent to the pericardium (red arrow)

Based on the results of previous experimental and clinical studies, NSCLC adjacent to the pericardium was defined as index tumors within a distance of 1.0 cm from the pericardium, according to CT scans [18]. Axial images were evaluated using a picture archiving and communication system under magnification. The tumors were classified into two groups, according to their distance from the heart: group A comprised tumors at a distance of 1–10 mm (i.e., tumors that were close but not contiguous with the pericardium), and group B included tumors at a distance of 0 mm (i.e., tumors that were contiguous with the pericardium) (Fig. 2).

### Surgical Procedure

All surgeries were performed with curative intent and a goal of negative oncologic margins. Surgical approach (muscle-sparing thoracotomy or video-assisted thoracoscopic surgery) was chosen based on tumor depth and location: 85 patients underwent video-assisted thoracoscopic surgery, while the remaining 70 patients underwent thoracotomy. However, five patients scheduled for thoracoscopic resection underwent thoracotomy due to the presence of tenacious pleural adhesions and failure to locate the nodule during thoracoscopy.

### MWA Procedure

The treatment plan was designed through CT images, in which the location‐coordinate scale of CT was longitudinally adhered to the surface of the tumor. The treatment plan included: (1) to determine the location, size, shape, and relation to the organs near the lesion, (2) to position the punctured areas on the body surface, (3) to determine the best entry route from the puncture point to the deepest margin of the lesion (“target skin distance”). After successful anesthetization, the antenna (17 G, 18 cm water-cooled antenna) was positioned into the deepest margin of the lesion according to the preoperative-planned route. The antenna routes were discussed and selected in a way that avoids the intercostal artery, pulmonary bullae, great bronchovascular bundles, and the pericardium. Each antenna insertion tract was positioned parallel to the pericardium. The electrodes were advanced into the tumor step by step along with the planned approach, and adjustments made to correct the path toward the targeted ablation zone if the electrode went off course; care was taken to ensure that the electrode did not pull out lung tissue during readjustment, minimizing the number of lung membrane or lobar fissure repeat punctures. MWA (2450 MHz MTC-3CA microwave generator; Vision Medical, Nanjing, China) could be carried out after connecting the cold-circulating pipes and pumps, linking the MWA antenna and MWA machine with a cable, turning on the ablation power in accordance with the preset conditions (generally selected 20–40 W, 6–8 min). The MWA antenna was extracted after ablating the “needle track.” Contrast-material-enhanced CT was routinely performed immediately after the ablation to evaluate technical success and examine possible complications. If viable residual tumors were identified, the ablation zone was re-ablated as previously described. After the ablation procedure, all patients were admitted for overnight observation, according to the routine care standards of our institution.

### Follow-Up

Clinical examination, complete blood cell count, and chest CT scan were performed the next morning in the MWA group to assess complications, such as pneumothorax, subcutaneous pneumoderma, alveolar hemorrhage, bleeding, and pleural effusion. When pericardial effusion was suspected (palpitations, fast regular rhythm, chest stuffiness, shortness of breath, dyspnea), echocardiography, cardiac ultrasound, or cross-sectional imaging with CT confirmed the diagnosis. Upon clinical suspicion of complications in patients in the WR group, CT imaging was conducted. After discharge, patients in both groups underwent a contrast-enhanced CT scan and a lung protocol at 1, 3, and 6 months after the procedure and subsequently after every six months. During follow-up, a PET/CT scan was performed, when the tumor activity cannot be identified by routine examination, such as the presence of inflammatory tissue surrounding the thermal injury, atelectasis, and tissue necrosis [19]. Additionally, the occurrence of distant (extra-cranial) metastasis was assessed by a PET scan. To rule out any subclinical cardiac injury, postprocedural myocardial enzyme levels (troponin test, creatine kinase test, and myoglobin) and echocardiograms were reviewed for changes from baseline.

### Study Outcomes

Local tumor progression (LTP) was defined as the new appearance of tumor at the margin of the ablation zone on follow-up images. Intrapulmonary distal recurrence (IDR) was defined as emergence of the recurrent tumor in the lung somewhere other than the area treated with MWA. Progression-free survival (PFS) was defined as the interval between initial treatment to tumor progression or death [20]. OS was defined as the interval between treatment and death or the date of last follow-up visit. Adverse events were recorded using the Common Terminology Criteria for Adverse Events [21]. General condition (evaluated six months after the treatment) was assessed according to the Karnofsky scale performance status [22]. Subgroup analysis of these outcomes was conducted according to the distance from the pericardium. Further, interaction effects between the treatment group and the distance from pericardium were examined for these outcomes.

### Statistical Analysis

For the entire data, continuous variables were analyzed using the two-sample *t* test if the assumption of normality was satisfied; otherwise, the Wilcoxon rank-sum test was used. Categorical variables were analyzed using the *χ*2 test. The effect of selection bias and confounding factors was reduced by calculating the propensity score using logistic regression and performing 1:1 patient matching [23]. The standardized mean difference was computed to assess the balance of variables used for matching and confirm whether the values were lower than 0.1. The cumulative recurrence rates for each type of disease (i.e., LTP, IDR) and survival rate were estimated with using the Kaplan–Meier method. Univariate and multivariate analyses were performed to determine the prognostic factors for, including clinical and biologic parameters for OS and PFS. All variables with the *P* value less than 0.05 in the univariate analyses were included in the multivariate analysis with Cox proportional hazards model. *P* value less than 0.05 was considered to indicate a significant difference. All statistical analyses were conducted with SPSS version 18 software (SPSS, Chicago, III).

## Results

### Baseline Characteristics

The baseline characteristics of all patients (*n* = 223) are presented in Table 1. The median follow-up period for the WR group was 48 months (range, 6–90 months) and 45 months (range, 4–86 months) in the MWA group (*P* = 0.952). Relative to patients in the WR group, patients in the MWA group were significantly older, more likely to have a poor lung function and shorter hospital stay. Although MWA patients had a worse baseline performance status with higher Charlson comorbidity index than surgical ones, no significant differences in other tumor characteristics were noted between the two groups.

**Table1:** Baseline characteristics of the study population

Characteristic or pulmonary functional parameter	WR group (*n* = 155)	MWA group (*n* = 68)	*P* value
Age, yr, mean (SD)	78.0 + − 11.8	83.1 + − 11.3	**0.002**
Female, *n* (%)	52 (33.5)	24 (35.3)	0.878
Baseline performance status	90 (80–100)	80 (60–90)	**0.001**
Charlson comorbidity index	3 (1–4)	5 (4–6)	**0.001**
Histology, *n* (%)	–	–	0.462
Adenocarcinoma	106 (68.4)	41 (60.3)	–
Squamous cell carcinoma	42 (27.1)	24 (35.3)	–
Other	7 (4.5)	3 (4.4)	–
Clinical stage, *n* (%)	–	–	0.23
T1aN0	62 (40.0)	21 (30.9)	–
T1bN0	93 (60.0)	47 (69.1)	–
Tumor size	2.5 (2.0–2.8)	2.3 (2.0–3.0)	0.117
Distance from pericardium	–	–	0.664
0	70 (45.2)	33 (48.5)	–
1–10	85 (54.8)	35 (51.5)	–
Neoplasms adjacent to	–	–	0.578
Left atrium	52 (33.5)	22 (32.4)	–
Left ventricle	14 (9.0)	8 (11.8)	–
Right atrium	43 (27.7)	23 (33.8)	–
Right ventricle	46 (29.7)	15 (22.1)	–
FVC, median, quartile range (%)	81 (66–97)	72 (64–92)	**0.001**
FEV1, median, quartile range (%)	71 (56–86)	63 (50–77)	**0.007**
DLCO, median, quartile range (%)	69 (54–82)	62 (49–74)	**0.02**
Post-treatment hospital stay days	6 (5–21)	2 (1–5)	**0.001**

Bold values indicate standard deviation (SD)

*WR* wedge resection, *MWA* microwave ablation, *FVC* forced vital capacity, *FEV1* forced expiratory volume in 1 s, *DLCO* diffusion capacity of the lung for carbon monoxide

No treatment-related deaths were recorded. However, nine patients (13.2%), developed major complications associated with MWA, and included pneumothorax (n = 5), pleural effusion (*n* = 2), and hemothorax (*n* = 2). We recorded 20 minor complications associated with MWA, including arrhythmia (*n* = 3), self-limited pneumothorax (*n* = 11), mild pericardial effusion (*n* = 3), and thickening of pericardial layers (*n* = 3). Major complications associated with WR occurred in thirty sessions (19.4%) and included chest tube required at discharge (*n* = 11), pneumonia (*n* = 13), empyema (*n* = 3), chylothorax (*n* = 4), and atrial fibrillation (*n* = 12).

### Comparison of Therapeutic Outcomes Before Propensity Score Matching

Local tumor progression (LTP) and intrapulmonary distant recurrence (IDR). During follow-up, LTP occurred in 8 of 155 WR patients (5.2%) and in 18 of 68 MWA patients (26.5%). The cumulative LTP rates at 1, 2, and 5 years were 4.0%, 5.0%, and 5.0% for the WR group and 13.0%, 23.0%, and 29.0%, respectively, for the MWA group (*P* < 0.001). IDR was identified in 48 WR patients (31.0%) and 23 MWA patients (33.8%). The cumulative IDR rates at 1, 2, and 5 years were 3.0%, 18.0%, and 31.0% for the WR group and 5.0%, 19.0%, and 35.0%, respectively, for the MWA group (*P* = 0.126).

**Fig. 3:**
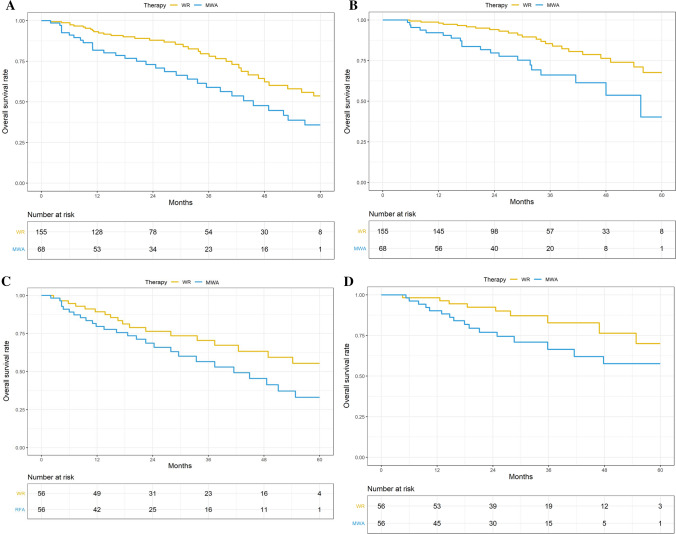
Types of recurrence and survival curves before and after matched patients from the wedge resection (WR) and microwave ablation (MWA) groups. **A** Progression-free survival rates. **B** Overall survival rates. **C** Progression-free survival rates. **D** Overall survival rates

PFS and OS. As of December 31, 2018, 23 of 155 WR patients (14.8%) and 20 of 68 MWA patients (29.0%) died. The 1-, 3-, and 5-year PFS rates were 92.0%, 74.0%, and 58.0% in the WR group and 82.0%, 57.0%, and 38.0% in the MWA group, respectively (*P* = 0.004) (Fig. 3A). The 1-, 3-, and 5-year OS rates were 98.0%, 84.0%, and 73.0% in the WR group and 92.0%, 63.0%, and 55.0% in the MWA group, respectively (*P* < 0.001) (Fig. 3B).

### Comparison of Therapeutic Outcomes After Propensity Score Matching

To compare therapeutic outcomes we matched 56 patients in each group and found that the baseline characteristics were well-balanced between the two groups (Table 2). However, baseline performance status (*P* = 0.011), Charlson comorbidity index (*P* = 0.015), and percentage of predicted forced vital capacity (FVC%) predicted value (*P* = 0.035) were not matched. Using the matched data, the cumulative LTP rates at 1, 2, and 5 years were approximately 4.0%, 5.0%, and 5.0% in the WR group and 11.0%, 22.0%, and 25.0% in the MWA group, respectively (*P* = 0.027). The cumulative IDR rates at 1, 2, and 5 years were 6.0%, 27.0%, and 36.0% for the WR group and 9.0%, 24.0%, and 38.0%, respectively, for the MWA group (*P* = 0.327). The PFS rates at 1, 3, and 5 years were 89.0%, 66.0%, and 56.0% in the WR group and 80.0%, 54.0%, and 36.0% in the MWA group, respectively (*P* = 0.029) (Fig. 3C). The estimated OS rates at 1, 3, and 5 years were 100.0%, 81.0%, and 72.0% in the WR group and 90.0%, 60.0%, and 55.0% in the MWA group, respectively (*P* = 0.047) (Fig. 3D).

**Table 2:** Baseline characteristics of study patients before and after propensity score analysis

Characteristic	Before propensity score matching			After propensity score matching		
	WR group (*n* = 155)	MWA group (*n* = 68)	*P* value	WR group (*n* = 56)	MWA group (*n* = 56)	*P* value
Age, yr, mean (SD)	78.0 + − 11.8	83.1 + − 11.3	**0.002**	78.8 + − 10.9	82.0 + − 9.8	0.112
Baseline performance status	90 (80–100)	80 (60–90)	**0.001**	90 (70–100)	80 (70–90)	**0.011**
Charlson comorbidity index	3 (1–4)	5 (4–6)	**0.001**	4(2–5)	4 (3–5)	**0.015**
FVC, median, quartile range (%)	81 (66–97)	72 (64–92)	**0.001**	80 (65–88)	75 (56–85)	**0.035**
FEV1, median, quartile range (%)	71 (56–86)	63 (50–77)	**0.007**	67 (53–79)	65 (46–75)	0.071
DLCO, median, quartile range (%)	69 (54–82)	62 (49–74)	**0.02**	70 (52–82)	63 (51–74)	0.139

Bold values indicate standard deviation (SD)

*WR* wedge resection, *MWA* microwave ablation, *FVC* forced vital capacity, *FEV1* forced expiratory volume in 1 s, *DLCO* diffusion capacity of the lung for carbon monoxide

### Analysis of Risk Factors for Therapeutic Outcomes

Multivariate analysis of all study patients (*n* = 223) showed MWA (hazard ratio [HR], 2.26; 95% CI, 1.52–3.35; *P* < 0.001), distance from pericardium (HR, 0.318; 95% CI, 0.166–0.608; *P* = 0.001), and tumor size (HR, 2.045; 95% CI, 1.239–3.374; *P* = 0.005) were significant factors for poor PFS (Table 3). With respect to OS, T1bN0 stage (HR, 0.506; 95% CI, 0.264–0.971; *P* = 0.04), distance from pericardium (HR, 0.192; 95% CI, 0.090–0.412; *P* = 0.001), and tumor size (HR, 2.024; 95% CI, 1.038–3.948; *P* = 0.039) were independent prognostic factors for a poor OS (Table 4).

**Table 3:** Risk factor analysis for progression-free survival

Factor	Univariate analysis		Multivariate analysis	
	Hazard ratio	*P* value	Hazard ratio	*P* value
Age, yr, mean (SD)	1.016 (0.993, 1.040)	0.176	…	…
Male, *n* (%)	0.985 (0.598, 1.622)	0.951	…	…
Charlson comorbidity index	1.087 (0.943, 1.253)	0.248	…	…
Histology, *n* (%)	0.492 (0.273, 0.887)	**0.018**	0.873 (0.430, 1.773)	0.707
Adenocarcinoma	–	–	–	–
Squamous cell carcinoma	–	–	–	–
Other	–	–	–	–
Clinical stage (T1bN0)	0.416 (0.254, 0.682)	**0.001**	1.643 (0.488, 5.528)	0.257
Tumor size	2.766 (1.685, 4.541)	**0.001**	2.045 (1.239, 3.374)	**0.005**
Distance from pericardium (0)	0.243 (0.138, 0.428)	**0.001**	0.318 (0.166, 0.608)	**0.001**
Neoplasms adjacent to atrium/ventricle	0.854 (0.609, 1.198)	0.361	…	…
Treatment type (MWA)	2.051 (1.264, 3.326)	**0.004**	2.240 (1.311, 3.829)	**0.003**
FVC, median, quartile range (%)	0.979 (0.964, 0.994)	**0.006**	0.997 (0.980, 1.015)	0.751
FEV1, median, quartile range (%)	0.981 (0.964, 0.997)	**0.024**	0.994 (0.977, 1.012)	0.532
DLCO, median, quartile range (%)	0.989 (0.965, 1.015)	0.41	…	…

Bold values indicate standard deviation (SD)

*WR* wedge resection, *MWA* microwave ablation, *FVC* forced vital capacity, *FEV1* forced expiratory volume in 1 s, *DLCO* diffusion capacity of the lung for carbon monoxide

**Table 4:** Risk factor analysis for overall survival

Factor	Univariate analysis		Multivariate analysis	
	Hazard ratio	*P* value	Hazard ratio	*P* value
Age, yr, mean (SD)	0.995 (0.969, 1.022)	0.734	…	…
Male, *n* (%)	0.946 (0.508, 1.763)	0.862	…	…
Charlson comorbidity index	1.100 (0.927, 1.306)	0.273	…	…
Histology, *n* (%)	0.677 (0.353, 1.297)	0.239	…	…
Adenocarcinoma	–	–	–	–
Squamous cell carcinoma	–	–	–	–
Other	–	–	–	–
Clinical stage (T1bN0)	0.296 (0.156, 0.560)	**0.001**	0.506 (0.264, 0.971)	**0.04**
Tumor size	2.157 (1.089, 4.273)	**0.028**	2.024 (1.038, 3.948)	**0.039**
Distance from pericardium (0)	0.208 (0.102, 0.424)	**0.001**	0.192 (0.090, 0.412)	**0.001**
Neoplasms adjacent to atrium/ventricle	0.902 (0.736, 1.138)	0.571	…	…
Treatment type (MWA)	2.291 (1.363, 4.372)	**0.008**	1.432 (0.698, 2.953)	0.237
FVC, median, quartile range (%)	0.863 (0.454, 1.256)	0.368	…	…
FEV1, median, quartile range (%)	0.902 (0.834, 1.012)	0.475	…	…
DLCO, median, quartile range (%)	0.989 (0.965, 1.015)	0.594	…	…

Bold values indicate standard deviation (SD)

*WR* wedge resection, *MWA* microwave ablation, *FVC* forced vital capacity, *FEV1* forced expiratory volume in 1 s, *DLCO* diffusion capacity of the lung for carbon monoxide

**Table 5:** Subgroup analysis according to the distance from pericardium

Variable	Outcome	The distance classification from pericardium	HR (95% CI)	*P* value	*P* value for interaction
Treatment type [local ablation]	LTP	Lesions that were contiguous to the pericardium (*n* = 33)	2.15 (1.14, 3.88)	0.011	*P* = 0.018
		Lesions that were not contiguous to the pericardium (*n* = 35)	1.62 (1.28, 2.65)	0.015	
	IDR	Lesions that were contiguous to the pericardium (*n* = 33)	0.57 (0.24, 1.65)	0.132	*P* = 0.246
		Lesions that were not contiguous to the pericardium (*n* = 35)	1.43 (0.66, 3.04)	0.365	
	PFS	Lesions that were contiguous to the pericardium (*n* = 33)	1.44 (1.23, 2.27)	0.013	*P* = 0.025
		Lesions that were not contiguous to the pericardium (*n* = 35)	1.52 (0.96, 2.59)	0.082	
	OS	Lesions that were contiguous to the pericardium (*n* = 33)	2.36 (1.35, 4.02)	0.012	*P* = 0.082
		Lesions that were not contiguous to the pericardium (*n* = 35)	1.46 (0.31, 2.18)	0.192	

*LTP* local tumor progression, *IDR* intrapulmonary distal recurrence, *PFS* progression-free survival, *OS* overall survival

### Subgroup Analysis by the Distance from the Pericardium

For the cases of NSCLCs that were contiguous to the pericardium, LTP, PFS, and OS were better in the WR than in the MWA group (*n* = 33, both *P* values < 0.05). According to the treatment modality, IDR (*P* = 0.365) and OS (*P* = 0.192) were not significantly different in patients with NSCLCs that were close but not contiguous to the pericardium. Significant interaction effects between the treatment group and distance from pericardium were observed for LTP and PFS (*P* = 0.018 and *P* = 0.025, respectively) (Table 5).

## Discussion

New remedies are currently being developed to treat early-stage primary lung malignancy in an ever-increasing population of inoperable and high-risk patients. The present study demonstrated that both WR and image-guided MWA can achieve satisfactory oncological results for the treatment of stage I NSCLC adjacent to the pericardium. While WR was superior in local control of the tumor, patients treated by MWA experienced lower invasiveness, less procedure-related adverse events, and a shorter hospital stay relative to WR. No significant differences were achieved in distant recurrence and overall survivals for both treatments. Based on the results of our multivariable analysis, treatment type did not significantly affect the long-term therapeutic outcomes for stage I NSCLC adjacent to the pericardium.

In our study, PFS rates were different between the two treatments, regardless of the propensity score analysis. Among the tested PFS factors, significant differences between the two groups were noted in intrapulmonary local recurrence, whereas no significant intergroup differences were observed in distant intrapulmonary recurrence. These results concur with the findings of previous studies [16, 17]. Possible explanations for the outcome are that the location, for a tumor adjacent to the pericardium, could increase the technical difficulty of adequately placing the electrodes, resulting in more frequent use of hydrodissection and overlapping ablation, compared with tumors that were contiguous to the pericardium [18, 24]. Further, the poor lung function state in the MWA group was adjusted by matched analysis because the condition could have increased the risk of pulmonary complications after the puncture. In stage I NSCLC patients, Ambrogi MC et al. reported a local recurrence rate of 2 and 23% (*P* = 0.002) following wedge resection and RFA, respectively [16].

In subgroup analysis, according to tumor distance from the pericardium, PFS and OS in patients with tumors contiguous to the pericardium were significantly better in the WR group than in the MWA group. However, no significant differences were observed in PFS and OS in patients with tumors not contiguous to the pericardium. These results suggest a relatively increased risk of LTP when performing MWA for tumors contiguous to the pericardium. Notably, this increased risk may affect the survival outcome. Although intrapulmonary recurrence in clinical treatment can be controlled with subsequent treatments [25], occurrence of extrapulmonary metastasis compromises on an effective therapy for tumor control [26]. Our results suggest that MWA can be considered as an alternative option for treatment of inoperable stage I NSCLC patients with tumors that are not contiguous to the pericardium. In contrast, WR should be preferred over MWA for stage I NSCLC patients with tumors contiguous to the pericardium.

The goal of treatment for stage I NSCLC is improvement of patient survival. Therefore, therapies should be selected based on strong evidence of efficacy, such as randomized controlled trials (RCTs) [27]. However, RCTs that can suggest an option for optimal treatment of stage I NSCLC adjacent to the pericardium are currently limited. Our findings suggest that tumor location relative to the pericardium and lung function condition needs to be considered in the choice of either WR or MWA for treatment of stage I NSCLC adjacent to the pericardium. Although the MWA strategy applied in this study has been used as a mature interventional therapy for high-risk and inoperable lung cancer patients [28, 29], the combined treatment with transcatheter pulmonary bronchial artery chemoembolization and microwave ablation [30, 31] might be reasonable alternatives for stage I NSCLCs contiguous to the pericardium. However, more studies are needed to evaluate the effectiveness of these treatments.

This study had several limitations. First, we used a retrospective approach. Our study is, therefore, fundamentally flawed by selection and indication bias. Due to the retrospective nature of the study, patients undergoing MWA were older, with lower baseline characteristics, and higher comorbidity score, since they were medically inoperable. Second, although we conducted a propensity score matching and multivariate analysis to enhance intergroup comparison, several unidentified biases may have favored the WR group. Third, evaluation of the suitable type of surgical approach (thoracotomy or thoracoscopic resection) is a potential factor when choosing the treatment modality. Finally, our study lacks a direct comparison with a cohort of patients treated with stereotactic body radiation therapy since the correlation was assessed using data from literature.

## Conclusion

In conclusion, WR provided better long-term tumor control and OS compared to MWA for stage I NSCLC adjacent to the pericardium as a first-line treatment. The present study supports that MWA can be considered as an alternative option for high-risk and inoperable patients, particularly for tumors that were not contiguous to the pericardium. Clinicians should evaluate preoperative baseline characteristics and tumor distance from pericardium when balancing the risk benefit of first-line treatment for early-stage lung cancer adjacent to the pericardium.
